# High Skp2 expression is associated with a mesenchymal phenotype and increased tumorigenic potential of prostate cancer cells

**DOI:** 10.1038/s41598-019-42131-y

**Published:** 2019-04-05

**Authors:** Šárka Šimečková, Zuzana Kahounová, Radek Fedr, Ján Remšík, Eva Slabáková, Tereza Suchánková, Jiřina Procházková, Jan Bouchal, Gvantsa Kharaishvili, Milan Král, Petr Beneš, Karel Souček

**Affiliations:** 1 Department of Cytokinetics, Institute of Biophysics of the Czech Academy of Sciences, Brno, Czech Republic; 2 Center of Biomolecular and Cellular Engineering, International Clinical Research Center, St. Anne´s University Hospital Brno, Brno, Czech Republic; 3 Department of Experimental Biology, Faculty of Science, Masaryk University, Brno, Czech Republic; 4 Department of Chemistry and Toxicology, Veterinary Research Institute, Brno, Czech Republic; 5 Department of Clinical and Molecular Pathology, Institute of Molecular and Translational Medicine, Faculty of Medicine and Dentistry, Palacky University, Olomouc, Czech Republic; 6 Department of Urology, University Hospital, Olomouc, Czech Republic; 7 Present Address: Human Oncology & Pathogenesis Program, Memorial Sloan Kettering Cancer Center, New York, New York, 10065 USA

## Abstract

Skp2 is a crucial component of SCF^Skp2^ E3 ubiquitin ligase and is often overexpressed in various types of cancer, including prostate cancer (PCa). The epithelial-to-mesenchymal transition (EMT) is involved in PCa progression. The acquisition of a mesenchymal phenotype that results in a cancer stem cell (CSC) phenotype in PCa was described. Therefore, we aimed to investigate the expression and localization of Skp2 in clinical samples from patients with PCa, the association of Skp2 with EMT status, and the role of Skp2 in prostate CSC. We found that nuclear expression of Skp2 was increased in patients with PCa compared to those with benign hyperplasia, and correlated with high Gleason score in PCa patients. Increased Skp2 expression was observed in PCa cell lines with mesenchymal and CSC-like phenotype compared to their epithelial counterparts. Conversely, the CSC-like phenotype was diminished in cells in which *SKP2* expression was silenced. Furthermore, we observed that Skp2 downregulation led to the decrease in subpopulation of CD44^+^CD24^−^ cancer stem-like cells. Finally, we showed that high expression levels of both CD24 and CD44 were associated with favorable recurrence-free survival for PCa patients. This study uncovered the Skp2-mediated CSC-like phenotype with oncogenic functions in PCa.

## Introduction

Prostate cancer is the second leading cause of cancer-related deaths in men in western countries^1^. Resistance to conventional treatments and the development of castration-resistant prostate cancer remain challenges of current prostate cancer therapies. The need for identification of new targets to treat this disease is therefore tremendous.

The epithelial-to-mesenchymal transition (EMT) is a physiological process during embryogenesis that may become reactivated in cancer. It is characterized by the loss of cell-to-cell adhesion and apical-basal polarity, and the gain of migratory behaviour^2^. EMT has been described as a crucial step in the progression and metastasis of prostate cancer^3^. Furthermore, the acquisition of a mesenchymal phenotype, concomitant with a cancer stem cell (CSC) phenotype, in prostate cancer has been shown previously^4–6^. EMT and CSCs play important roles in the development of drug resistance in cases of prostate cancer^7^.

CSCs have been described as a subset of cells within a heterogeneous tumor that share a number of features with normal stem cells. CSCs are characterized by self-renewal, the expression of specific surface markers, and aldehyde dehydrogenase (ALDH) activity^8,9^. CSCs are also involved in tumor initiation, metastasis, and chemoresistance^10^. The CSC marker CD24 has been described as a marker that distinguishes poorly differentiated cells from transit-amplifying cells in the basal layer of the human prostate^11^. Cells with a CD24^−^CD44^+^ phenotype are commonly used to define prostate CSCs^12,13^.

The cyclin-dependent kinase inhibitor p27^Kip1^ was shown to control both stem cell renewal and EMT in embryonic stem cells^14^. Importantly, S-phase kinase-associated protein 2 (Skp2) is the main regulator of p27^Kip1^ protein stability^15,16^. High expression of Skp2 in tumors, accompanied by p27^Kip1^ downregulation, has been correlated with poor prognosis in cancer patients; Skp2 has also been implicated as a prognostic marker in many types of cancer, including prostate cancer^17,18^. Skp2 is a variable component of SCF^Skp2^ (Skp, Cullin, F-box containing complex) E3 ubiquitin ligase, which is responsible for recognizing many substrates that are targeted for degradation in the proteasome^19^. The mechanisms that control Skp2 expression are not fully understood^20^. In prostate cancer, putative regulatory mechanisms of Skp2 include those involving the androgen receptor^21^, PTEN^17^, and PI3K/Akt^22^. In mice, an essential role of Skp2 in the development of prostate cancer was described as overexpression of Skp2 in the prostate gland induced hyperplasia, dysplasia, and low-grade carcinoma^23^. Conversely, Skp2 inactivation, together with senescence-induced oncogenic stress, was shown to profoundly restrict tumorigenesis *in vivo*^24^.

The roles of Skp2 in both EMT and cancer stem-like cells in prostate cancer have not been fully elucidated. Herein, we show an association between elevated Skp2 levels and an EMT phenotype in patients with prostate cancer as well as in prostate cancer cell lines. Moreover, a mesenchymal phenotype was found to be associated with characteristics of cancer stem-like cells, including a DU 145 cell phenotype that was altered after Skp2 downregulation.

## Material and Methods

### Chemicals

DMSO (Sigma-Aldrich, St. Louis, MO, USA); 0.05% Trypsin-EDTA (GE Healthcare Life Sciences, Little Chalfont, UK); 1% BSA (Serva, Germany) in PBS with 0.01% NaN_3_ (Sigma-Aldrich); 0.25% Triton X-100 (Sigma-Aldrich) solution in PBS; 4% Paraformaldehyde solution (Sigma-Aldrich) in PBS; Aldehyde dehydrogenase-based cell detection kit (StemCell 01700); Fumitremorgin C (Abcam, ab144258); For detail specification regarding used antibodies - see Supplementary Tables S1–S3).

### Cell culture

DU 145 cells were obtained from ATCC and cultivated in RPMI 1640 (Thermo Fisher Scientific, TFS, Waltham, MA, USA) with 10% of fetal bovine serum (FBS; TFS), penicillin (100 U/ml) and streptomycin (0.1 mg/ml; Biosera, Nuaille, France). The epithelial and mesenchymal sublines were established from sorted single cell clones derived from Trop-2 positive or Trop-2 negative populations, as shown in Remsik *et al*.^25^. PC-3 cells were cultivated in Ham’s F12 media (TFS) with 10% of FBS, penicillin and streptomycin. E2 and cE2 murine prostate adenocarcinoma cells^26^ (a generous gift from Dr. Pradip Roy Burman, University of Southern California, Los Angeles, CA), were cultivated as described in^26^. PC-3 AC and PC-3 DR12 cells were obtained from prof. Watson (Dublin, Ireland) and cultivated as described^27^.

All cell lines were cultivated in TPP (Trasadingen, Switzerland) flasks and plates in a humidified incubator at 37 °C in an atmosphere of 5% CO_2_. The cells were harvested by washing with 0.05% EDTA in PBS followed by trypsinization (0.05% w/v trypsin/0.53 mM Trypsin-EDTA). The AmpFLSTR® Identifiler® PCR Amplification Kit (TFS) was used to verify the origin of cell lines.

### Electrophoresis and western blotting

The procedure was performed as described previously^28^. Antibodies used for western blot are listed in Supplementary Table S1.

### RNA isolation and qPCR analysis

Total RNA was isolated using a High Pure RNA Isolation Kit (Roche) and equal amounts of RNA were reverse transcribed using a High Capacity RNA-to-cDNA Kit (Life Technologies). Quantitative RT-PCR was carried out as described previously^28^ using TaqMan assays with primers and conditions specified in Supplementary Table S6. Relative changes in gene expression were calculated using the E-method based on real efficiency values obtained from calibration curves^29^.

### Preparation of *SKP2* KD cell lines

DU 145 were transfected with Skp2 p45 CRISPR/Cas9 KO Plasmid (h) (sc-400534) and Skp2 p45 CRISPR/Cas9 KO Plasmid HDR (sc-400534) using Lipofectamine 3000 (TFS) as recommended to prepare *SKP2* KD cell lines or with Control CRISPR/Cas9 Plasmid (sc-418922, all SCBT) and empty vector pIRES puro2 (kindly provided by V. Bryja, Masaryk University, Brno, Czech Republic) to prepare control cells. Cells were selected in media with puromycin (300 ng/ml; TFS) for one week. RFP positive single cells (indicating insertion of the plasmid with puromycin resistance in a site of CRISPR deletion) were sorted using FACSAria II Sorp system using a 100-μm nozzle (20 psi) to obtain single cell-derived *SKP2* KD clones. To prepare control cell lines, cells underwent the same procedure as *SKP2* KD cells. Therefore, viable single cells were sorted. Post-sorting purity was determined immediately after sorting. The protein level of Skp2 in *SKP2* KD and control cells was examined by western blot.

### Spheroids formation assay

For spheroid formation assay, cells were seeded in semisolid media (0.1% agarose in complete culture media) on plates precoated with 0.5% agar and cultured for three weeks. Cells were seeded in low density, 500 cells/well in a 6-well plate. Spheroids were stained with MTT^30^ and counted using ImageJ software (NIH). For analysis of cancer stem cells markers in 3D, cells were seeded in high density (10 000 cells/well in 6 well plate) in semisolid media (0.1% agarose in complete culture media) on plates precoated with 0.5% agar and cultivated for three weeks. Spheroids were harvested, incubated with prewarmed PBS/EDTA and Trypsin to obtain a single cell suspension. Cells were then stained with antibodies according to standard procedure and analyzed on a flow cytometer.

### Tumorsphere formation assay

For tumorsphere formation assay, cells were seeded in low density (500 cells per well) in stem cell growth medium in ultra-low attachment 6 well plates (Corning #3471) and were grown for two weeks. After two weeks, tumorspheres were stained with MTT^30^ and were analysed and counted using ImageJ software (NIH).

### Flow cytometry analysis

Sample acquisition was performed with using FACSVerse (BD Biosciences). Flow cytometric data were analyzed using FlowJo software (Version 10.0.7, Tree Star, Ashland, OR, USA). Details about antibodies used are listed in Supplementary Table S2. Immunophenotype analysis was performed as described previously^31^. Cells were harvested using PBS/EDTA and Trypsin and stained with particular antibodies or probes. Cell cycle analysis was performed as described previously^21^. ALDH1 activity was analyzed according to manufacturer’s protocol and as described previously^32^. Dead cells, aggregates, and debris were excluded from analysis.

### Cell painting and morphology analysis

The cells were seeded in density 50 000 cells/cm^2^ into 384 black well plate (4 wells per clone, Falcon, #353962) and grown for 2 days at standard conditions. Next, cells were stained with Hoechst 33342, concanavalin A, SYTO14, phalloidin, wheat germ agglutinin and MitoTracker as described previously^33^ with using epMotion 5075 (Eppendorf) liquid handling workstation. Images were acquired with using ImageXpress Micro (Molecular Devices) fluorescence microscope (40x objective). Briefly, 5 fluorescence channels – using DAPI, Cy3, GFP, TxRed, Cy5 filters were captured from 35 sites in each well or 1000 cells per well were acquired with adaptive acquisition set up. Spillovers of fluorescence from different channels were compensated by subtraction of dim signal based on single stained compensation controls. Representative images were artificially colored and scale bar was burned into images using ImageJ 1.51p (NIH). Acquired images were processed based on the original cell painting protocol^33^. Objects of nuclei, cytoplasm and whole cell areas were segmented and together 1785 features were calculated from 5 fluorescence channels on these three objects with using CellProfiler v2.2.0 software^34^. Data were scaled, missing data and constant values in specific features were removed before principal component analysis and t-SNE analysis (perplexity 1605, nr. of iterations 500, minimum cost value 0.5) were performed. Plots were produced in R (RStudio, Inc., Boston, MA, USA).

### Tumorigenic potential of SKP2 KD cell lines

The colony of severe combined immunodeficient animals (Crl:SHO-PrKD^scid/Hrhr^) was acquired from Charles River (Sulzfeld, Germany) and maintained according to the ARRIVE guidelines. Cells were injected into the right flank (1 × 10^6^ cells/mice; 10 mice per group) and six weeks after injection, mice were sacrificed. Tumour formation rate was then calculated for both control and SKP2 KD cells. The rate was calculated as a ratio of a number of mice inoculated with cancer cell and the number of mice bearing tumours. All procedures involving animals were performed according to the EU Directive 2010/63/EU for animal experiments and approved by the Academy of Sciences of the Czech Republic (AVCR 2013/13); supervised by the local ethical committee of the Institute of Biophysics of the Czech Academy of Sciences and performed by certified individuals.

### Immunostaining of clinical samples

Retrospective analysis of tissue samples was performed in three independent sets of patients with prostate carcinoma. The main set included 187 tissue samples (33 samples of BPH, 101 samples of PCa, 35 samples of seminal vesicles and 18 positive lymph nodes). Samples were examined for expression of Skp2, E-Cadherin, and vimentin. The study was approved by the Ethics Committee of the University Hospital and Medical Faculty of Palacky University in Olomouc. Immunostaining was performed with validated antibodies after appropriate antigen retrieval (Supplementary Table S3). The specimens were assessed semi-quantitatively using the histoscore, considering the percentage of positive cells (0–100%) multiplied by staining intensity (0–3), which results in a final histoscore between 0 and 300.

### Skp2 overexpression analysis

Cells were transfected using Neon transfection system (TFS) with control plasmid (pcDNA3.1) or pcDNA3-myc-Skp2 that was a gift from Axel Brunger (Addgene Plasmid #19947)^35^, incubated for two days and harvested for flow cytometric analysis to detect surface markers.

### Survival data analysis

Data were retrieved from^36^, (GEO accession no. GSE21032). Data for recurrence-free survival were re-analysed and plotted in Prism (v6, GraphPad, La Jolla, California, United States) using the log-rank Mantel-Cox test. The threshold for CD24 and CD44 was set to ‘low 25%’.

### Statistical analysis

Statistical analysis was performed using Prism (v6, GraphPad, La Jolla, California, United States) using Student´s t-test.

## Results

### High nuclear Skp2 expression and mesenchymal phenotype is associated with high Gleason score in patients with prostate cancer

**Figure 1:**
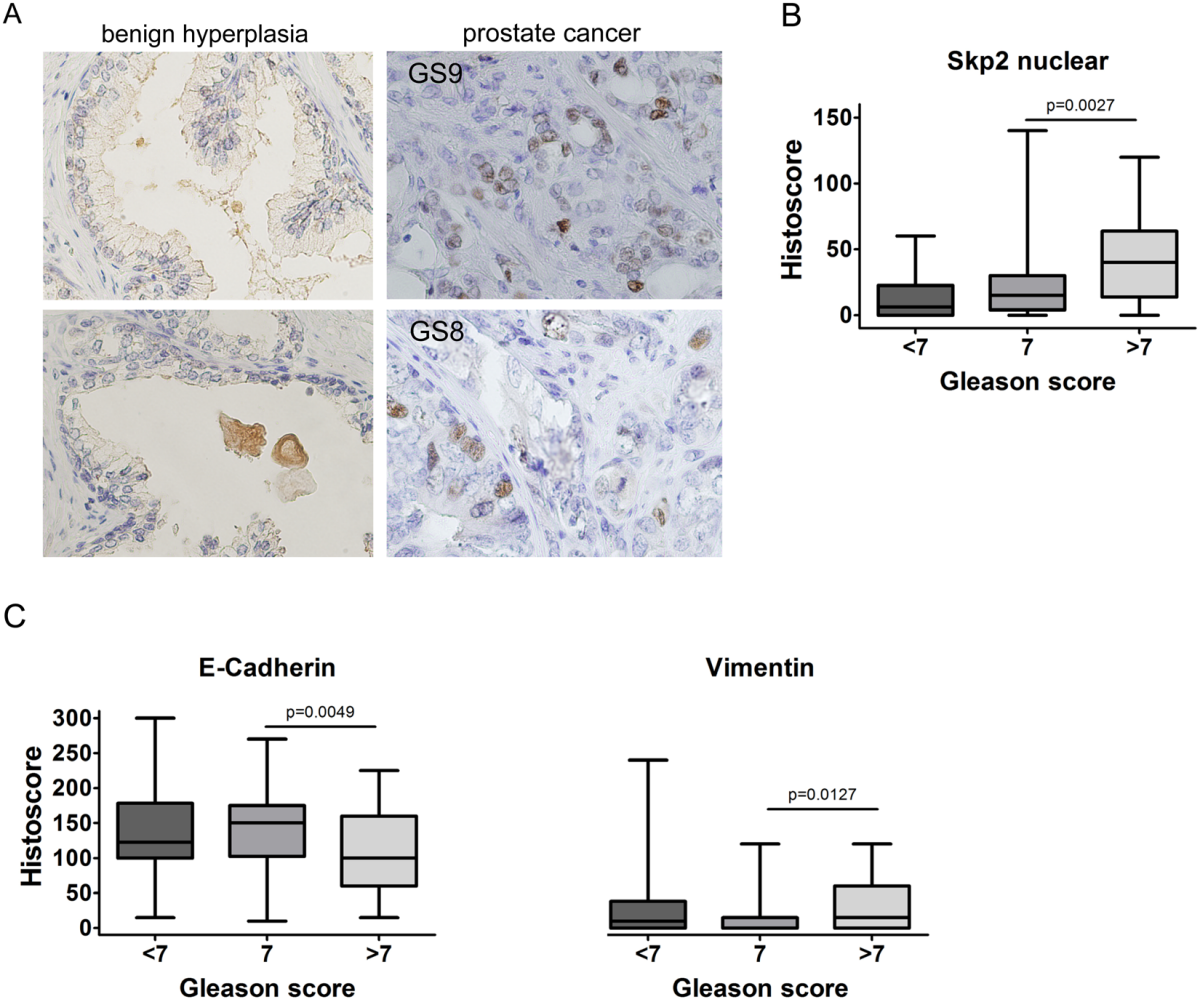
Nuclear Skp2 expression is associated with a high Gleason score in prostate cancer patients. (**A**) Patient samples from primary prostate carcinoma and benign hyperplasia were stained with anti-Skp2 antibody. Magnification: 40×. (**B**) Correlation of Skp2 with Gleason score. (**C**) Correlation of EMT-related marker (E-cadherin and vimentin) expression with Gleason score.

To confirm the role of Skp2 in prostate cancer progression, we performed a retrospective analysis of Skp2 expression in patients with benign hyperplasia and prostate carcinoma (Supplementary Tables S4 and S5). Our results show that the nuclear expression of Skp2 was significantly increased in prostate cancer tissues compared to benign hyperplasia tissues (Fig. 1A). High Gleason score (≥7) was associated with high nuclear, but not cytoplasmic, Skp2 expression (Fig. 1B and Supplementary Fig. 1). Moreover, a high Gleason score was associated with a mesenchymal phenotype, as demonstrated by decreased E-cadherin expression and increased vimentin expression (Fig. 1C). Thus, we showed that the nuclear expression of Skp2 is significantly increased in patients with a high Gleason score (≥7) whose cancer cells exhibit characteristics of the mesenchymal state.

### Increased Skp2 is associated with the mesenchymal phenotype of prostate cancer cells

**Figure 2:**
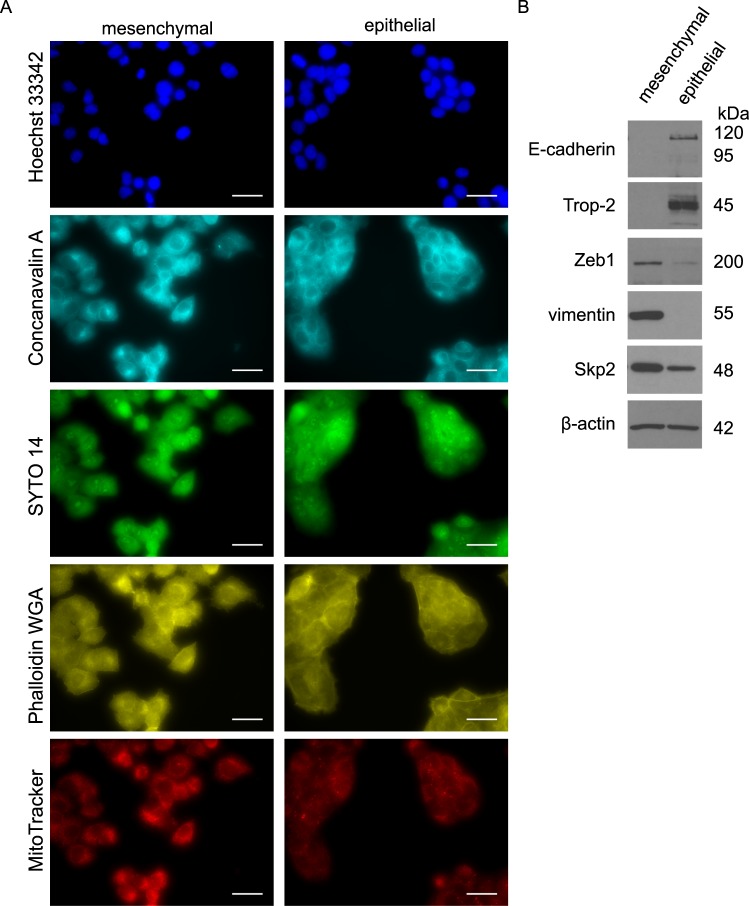
Increased Skp2 expression is associated with the mesenchymal phenotype of prostate cancer cells. (**A**) Representative images of mesenchymal and epithelial DU 145 sublines, scale bar size 30 µm. The nuclei (Hoechst 33342), endoplasmic reticulum (Concanavalin A), nucleoli and cytoplasmic RNA (SYTO 14), actin, Golgi apparatus, plasma membrane (Phalloidin&WGA) and mitochondria (MitoTracker) were visualized according to cell painting protocol. (**B**) Expression of mesenchymal and epithelial markers and Skp2 in sublines of DU 145 cells by western blot. Representative images are from three independent experiments. β-actin was used as a loading control.

To assess the connection between Skp2 expression and a mesenchymal phenotype in prostate cancer cells, we employed three sets of prostate epithelial cell lines and their mesenchymal counterparts. To verify the epithelial characteristics of these cells, we confirmed the expression of both E-cadherin and Trop-2, which belongs to the same gene family as does EpCAM, and whose overexpression has been described in various human epithelial cancers. The induction of mesenchymal phenotype was defined by the expression of vimentin, N-cadherin and transcription repressor of E-cadherin, ZEB1. Moreover, we documented the differences in morphology of mesenchymal and epithelial sublines using cell painting protocol^33^ and unsupervised image analysis (Fig. 2A, Supplementary Fig. 2A,B).

In the cell lines used, an EMT phenotype was induced by different processes. We compared human DU 145 epithelial and mesenchymal sublines (Fig. 2A,B, Supplementary Fig. 2A,B) that were previously prepared in our laboratory^37^. We then used age-control PC-3 cells (AC; epithelial) and docetaxel-resistant PC-3 cells (DR; mesenchymal), in which the acquisition of docetaxel resistance was accompanied by EMT^27^ (Supplementary Fig. 2C). Lastly, we employed mouse prostate adenocarcinoma cell lines with a biallelic *PTEN* depletion: cE2 (epithelial, androgen independent) and E2 (mesenchymal, androgen-dependent)^26,28^. We found that the mesenchymal phenotype of prostate cancer cells was accompanied by an increase in Skp2 levels in all selected pairs of cell lines (Fig. 2B, Supplementary Fig. 2C).

### Mesenchymal DU145 cells exhibit characteristics of cancer stem-like cells

**Figure 3:**
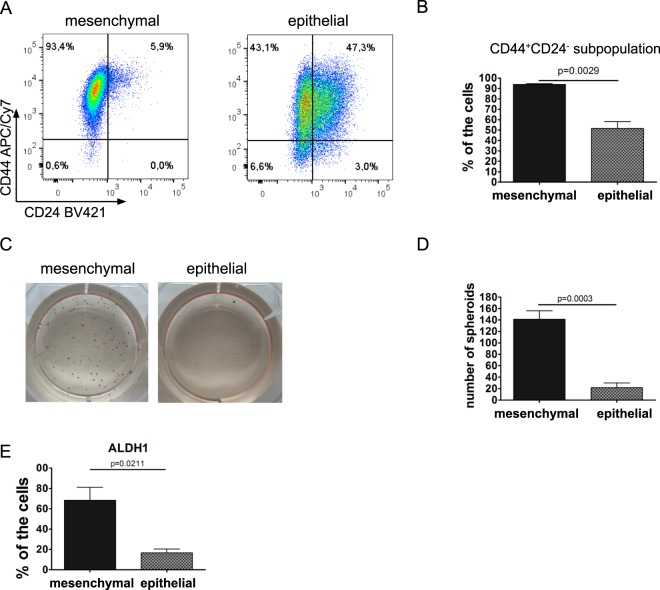
DU 145 cells with a mesenchymal phenotype are considered cancer stem-like cells. (**A**) Analysis of CD44 and CD24 markers in mesenchymal and epithelial DU 145 cells using flow cytometry. Dot plots show representative results from three independent experiments. (**B**) Quantitation of results shown in (**A**). Bars show the mean ± SD of the percentage of CD44^+^CD24^−^ cancer stem-like cells (n = 3). (**C**) Spheroid formation and growth in anchorage-independent conditions in DU 145 mesenchymal and epithelial cells. (**D**) Quantitation of results shown in (**C**). Data are shown as the mean ± SD of the number of spheroids per well. Results are from three independent repetitions. (**E**) ALDH1 activity in DU 145 mesenchymal and epithelial sublines. Data are presented as the mean ± SD of the percentage of cells with active ALDH1. Results are from three independent repetitions.

The properties of cancer stem-like cells were further investigated in epithelial and mesenchymal sublines established from DU 145 cells. We observed that mesenchymal cells are enriched in a CD44^+^CD24^−^ cancer stem cell subpopulation (Fig. 3A,B and Supplementary Fig. 3C). Downregulation of CD24 in mesenchymal cells was confirmed on both protein level (Supplementary Fig. 3A) and at the mRNA level (Supplementary Fig. 3B). To examine the CSC-like properties of the cells, epithelial and mesenchymal cells were seeded at low densities in semisolid media to examine their ability to grow in anchorage-independent conditions and form spheroids. The cancer stem-like cell phenotype of the mesenchymal cells was illustrated by their increased ability to form spheroids (Fig. 3C,D). This phenomenon was not caused by increased proliferation, as there were no differences in proliferation between mesenchymal and epithelial cells (Supplementary Fig. 3D). Further, the mesenchymal and epithelial sublines were seeded into a stem cell culture media and tumorsphere formation assay was performed. We observed a trend of increased tumorsphere formation rate in mesenchymal cells compared to epithelial cells (Supplementary Fig. 3E). Importantly, the size of the tumorspheres was significantly increased in mesenchymal cells (Supplementary Fig. 3F). The cancer stem-like properties of mesenchymal DU 145 cells were further confirmed by the increased activity of ALDH1 (Fig. 3E). Thus, we showed that DU 145 cells with a mesenchymal phenotype and high expression of Skp2 exhibit CSC characteristics compared to their epithelial counterparts.

### Decreased clonogenic and tumorigenic potential of DU 145 SKP2 KD cells is accompanied by decreased CD44^+^CD24^−^ CSC-like subpopulation

**Figure 4:**
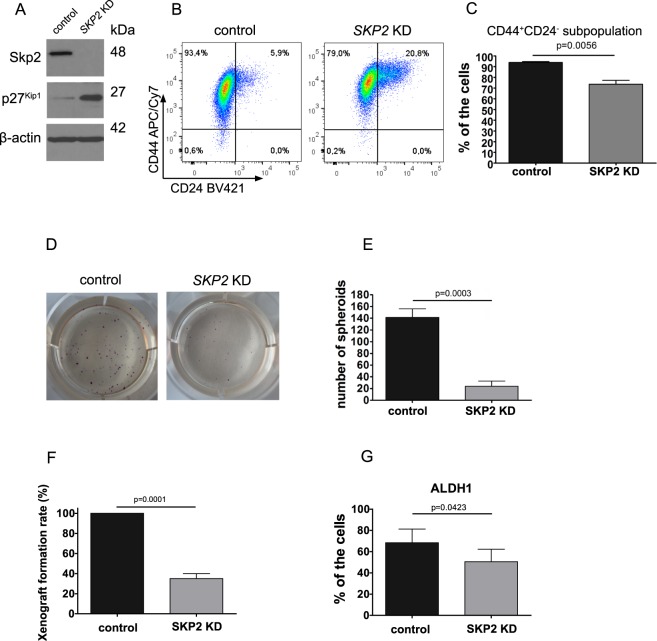
Decreased clonogenic and tumorigenic potential of mesenchymal DU 145 *SKP2* KD cells is accompanied by a decrease of CD44^+^CD24^−^ cancer stem cell subpopulation. (**A**) Skp2 and p27^Kip1^ expression in *SKP2* KD cells were determined by western blot. Representative images are from three independent experiments. β-actin was used as a loading control. (**B**) Analysis of CD44 and CD24 markers in control and *SKP2* KD cells using flow cytometry. Dot plots show representative results from three independent experiments. (**C**) Quantitation of results shown in (**B**). Data are presented as the mean ± SD of the percentage of CD44^+^CD24^−^ cancer stem-like cells (n = 3). (**D**) The capability of spheroid formation and growth in anchorage-independent conditions in *SKP2* KD cells and control cells. (**E**) Quantitation of results shown in (**D**). Data are shown as the mean ± SD of the number of spheroids per well. Results are from three independent repetitions. (**F**) The xenograft formation rate of *SKP2* KD cells in SHO mice. Tumour formation rate was then calculated for control and *SKP2* KD cells. A number of mice per group, n = 10. (**G**) ALDH1 activity in DU 145 mesenchymal cells with Skp2 knocked down. Data are presented as the mean ± SD of the percentage of cells with active ALDH1. Results are from three independent experiments.

To further ascertain the role of Skp2 in cancer stem-like cells, we decided to knock down Skp2 expression in DU 145 mesenchymal cells (DU 145 *SKP2* KD cells; Fig. 4A) and to analyze their tumorigenic capacity *in vitro* and *in vivo*. Silencing of Skp2 resulted in the accumulation of its substrate p27^Kip1^ (Fig. 4A). When detecting single markers, CD24^+^ and CD44^+^ cells were more abundant in *SKP2* KD (Supplementary Fig. 4A) and also mRNA levels were increased in *SKP2* KD cells (Supplementary Fig. 4B). However, when we investigated the size of CD44^+^CD24^−^ cancer stem-like subpopulation, we observed its significant downregulation (Fig. 4B,C and Supplementary Fig. 4A). We then examined the ability of these cells to grow in the anchorage-independent conditions and form spheroids. We found that the sphere-forming ability of *SKP2* KD cells was significantly decreased (Fig. 4D,E), whereas Skp2 knock-down itself did not impair cellular proliferation (Supplementary Fig. 4C). When we performed the tumorsphere assay, we observed significantly decreased tumorsphere size and formation rate in *SKP2* KD cells compared to control cells (Supplementary Fig. 4D,E). Next, *SKP2* KD cells were injected into immunodeficient mice, and the rate of xenograft formation was recorded. Our results showed that Skp2 knock-down significantly decreased the tumorigenic potential of DU 145 mesenchymal cells (Fig. 4F). Furthermore, the activity of ALDH1 was decreased in these cells (Fig. 4G). In conclusion, our data confirm that Skp2 silencing decreases the tumorigenic potential of mesenchymal DU 145 cells and affects the functional properties of cancer stem-like cells.

### Skp2 overexpression induces a decrease of CD24 in DU 145 but no change in CD44^+^CD24^−^

In our study, we observed a negative correlation between the decreased expression of Skp2 and the CD44^+^CD24^−^ phenotype in epithelial DU 145 cells compared to their mesenchymal counterparts (Figs 2B and 3A,B). Importantly, CD44^+^CD24^−^ subpopulation was decreased in DU 145 *SKP2* KD cells (Fig. 4B,C). Therefore, we hypothesized that Skp2 overexpression leads to increased CD44^+^CD24^−^ subpopulation in the DU 145 parental cell line. Indeed, in cells with transient Skp2 overexpression (Supplementary Fig. 5A), we observed a decrease in the percentage of CD24^+^ cells and a trend of increased percentage of CD44^+^CD24^−^ CSC-like subpopulation (Supplementary Fig. 5B). Thus, we showed that Skp2 is important for maintaining the cancer stem-like phenotype of DU 145 mesenchymal cells in term of the CD44^+^CD24^−^ phenotype.

**Figure 5:**
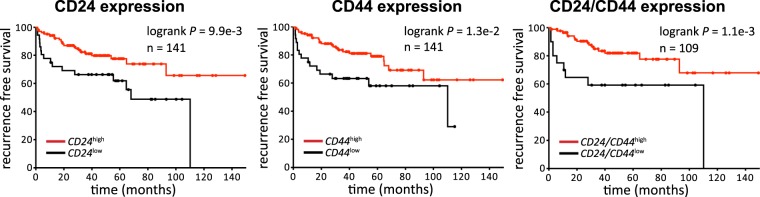
CD24 and CD44 expression is associated with better prognosis for prostate cancer patients. The graphs show an analysis of recurrence-free survival analysis in a cohort of prostate cancer patients, according to high or low CD24 (left), CD44 (middle), and CD24CD44 (right) expression levels. Data were retrieved from^36^. Data were plotted and reanalyzed using Prism (v6, GraphPad) using the log-rank Mantel-Cox test. The threshold for CD24 and CD44 was set to ‘low 25%’.

Our results suggest that the high expression of CD24 is linked to the epithelial and less aggressive phenotype of metastatic prostate cancer cells, accompanied by the low expression of Skp2. Conversely, low CD24 expression is linked to the mesenchymal phenotype of metastatic prostate cancer cells that have characteristics of cancer stem-like cells associated with high Skp2 expression. As Skp2 overexpression has been correlated with poor prognosis in prostate cancer patients, we wanted to reveal whether high CD24 expression would be associated with a better prognosis. To investigate the association of CD24 and CD44 expression with recurrence-free survival, we analyzed cohorts of prostate cancer patients^36^ and confirmed that high expression levels of both markers (CD24 and CD44) were associated with favourable recurrence-free survival (Fig. 5).

## Discussion

In our work, we showed that Skp2 expression was increased in cancerous prostate tissue compared to benign prostate tissue, specifically in tumours with a high Gleason score (≥7). Poorly differentiated tumours also had lower expression levels of E-cadherin and higher levels of vimentin, suggesting the ongoing EMT of prostate cancer cells.

Our results confirmed that Skp2 expression was increased in mesenchymal prostate cancer cells compared to their epithelial counterparts. Yet, the acquisition of the mesenchymal phenotype was associated with increased Skp2 expression in paclitaxel-resistant breast cancer cells^38^ and TGF-β1-induced EMT in melanoma cells^39^. Importantly, Ruan *et al*. revealed that Skp2 regulates progression of castration-resistant prostate cancer through Twist-mediated oncogenic functions including EMT and CSC acquisitions^6^.

We demonstrated that the mesenchymal state of DU 145 cells exhibited CSC properties: increased CD44^+^CD24^−^ subpopulation, increased ability to form spheroids, and high ALDH1 activity. These results are consistent with the acquisition of the CSC phenotype, which correlates with the mesenchymal state of the cancer cells. The CD44^+^CD24^−^ phenotype has been associated with CSCs, and the mesenchymal phenotype has been observed in several cancers, including breast cancer^40,41^, ovarian cancer^42^, and oral squamous carcinoma^43^. Similarly, prostate cancer cells with the CD44^+^CD24^−^ phenotype have been described as mesenchymal^44^. These results are in agreement with the work of Cremers *et al*. who showed that CD24 was not required for tumour initiation and growth in murine models of breast cancer and prostate cancer^45^. The mesenchymal state caused by docetaxel resistance in DU 145 and PC3 cells was linked to the acquisition of the CD44^+^CD24^−^ phenotype^4,5^. Further, it was shown that the overexpression of the mesenchymal-related genes *ZEB1* and *VIM* correlated with shorter radiologic progression, whereas low expression levels of CD24 correlated with shorter biochemical recurrence-free survival of patients with prostate cancer^5^.

To further assess the role of Skp2 in the tumorigenesis of the prostate cancer stem-like cells, we employed DU 145 cells in which *SKP2* had been knocked down. We confirmed that the knock-down of Skp2 significantly reduced the ability of these cells to form spheroids *in vitro* and decreased tumorigenic potential *in vivo*. Our findings are in agreement with the work of Lu *et al*. who found that Skp2 deficiency led to the suppression of prostate tumorigenesis^46^. These authors showed that Skp2 was involved in tumorigenesis via the repression of H3K4-specific demethylase JARID1B. Another study showed that Skp2 deficiency blocked tumorigenesis in the pRb/p53 double-deficient prostate tissue, suggesting that Skp2 was a promising target for drug treatment in pRb/p53-deficient tumours^47^. In addition to the decreased tumorigenicity of *SKP2* KD cells, we observed the decrease in percentage representation of CD44^+^CD24^−^ subpopulation and the decreased activity of ALDH1. These findings are in line with those of Zhao *et al*., where Skp2 downregulation or its pharmacological inhibition decreased the activity of ALDH1^47^.

Analysis of biochemical recurrence-free survival revealed that high expression levels of CD24 and CD44 were associated with a better prognosis for patients. These findings are in line with the work of Marin-Aguilera *et al*. who showed that low mRNA levels of CD24 correlated with shorter biochemical recurrence-free survival of patients with prostate cancer^5^. A decreased number of CD24-positive cells were also associated with the mesenchymal phenotype and DU 145 cell chemoresistance^5,48^. Furthermore, prostate cancer cells with low CD24 expression levels displayed high tumorigenic potential^49^, which is in line with our findings.

## Conclusion

In conclusion, we showed that the nuclear expression of Skp2 is associated with a high Gleason score. High Skp2 expression is further associated with the mesenchymal phenotype in prostate cancer patients and *in vitro*. Our results demonstrated that high expression levels of Skp2 were associated with the mesenchymal state and CSC characteristics. Lastly, we showed an inverse relationship between Skp2 expression and CD44^+^CD24^−^ cancer stem-like subpopulation, suggesting that Skp2 is a promising marker for prostate cancer susceptibility. In overall, we demonstrated the potential of Skp2 targeting in prostate cancer treatment.

## Supplementary information

Supplementary Materials
