# Supplementary material for: High Skp2 expression is associated with a mesenchymal phenotype and increased tumorigenic potential of prostate cancer cells
Source: Sci Rep. 2019 Apr 5;9:5695. doi: 10.1038/s41598-019-42131-y (PMC6451010; doi:10.1038/s41598-019-42131-y)

SUPPLEMEMTARY MATERIALS

High Skp2 expression is associated with a mesenchymal phenotype and increased tumorigenic potential of prostate cancer cells

Šárka Šimečková[1,2,3], Zuzana Kahounová[1,2], Radek Fedr[1,2], Ján Remšík[1,2,3*], Eva Slabáková[1], Tereza Suchánková[1], Jiřina Procházková[4], Jan Bouchal[5], Gvantsa Kharaishvili[5], Milan Král[6], Petr Beneš[2,3], Karel Souček[1,2,§]

[1]Department of Cytokinetics, Institute of Biophysics of the Czech Academy of Sciences, Brno, Czech Republic

[2]Center of Biomolecular and Cellular Engineering, International Clinical Research Center, St. Anne´s University Hospital Brno, Brno, Czech Republic

[3]Department of Experimental Biology, Faculty of Science, Masaryk University, Brno, Czech Republic

[4]Department of Chemistry and Toxicology, Veterinary Research Institute, Brno, Czech Republic

[5]Department of Clinical and Molecular Pathology, Institute of Molecular and Translational Medicine, Faculty of Medicine and Dentistry, Palacky University, Olomouc, Czech Republic

[6]Department of Urology, University Hospital, Olomouc, Czech Republic

*Current address: Human Oncology & Pathogenesis Program, Memorial Sloan Kettering Cancer Center, New York, New York 10065, USA

[§]Address for Correspondence: Karel Souček, Ph.D., Institute of Biophysics of the Czech Academy of Sciences, Královopolská 135, CZ 612 65 Brno, Czech Republic; Tel.: +420 541 517 166; E-mail: ksoucek@ibp.cz

Supplementary Figure Legends

Supplementary Figure 1. Cytoplasmic Skp2 expression does not correlate with a high Gleason score (≥ 7) in patients with prostate cancer. Data were plotted, reanalyzed using Prism (v6, GraphPad), and statistics was calculated using the Student's t-test.

Supplementary Figure 2.
(A) Principal component analysis and t-SNE analysis from 1761 morphology parameters of mesenchymal and epithelial DU 145 cells. t-Sne plot shows different phenotype based on the morphology of mesenchymal and epithelial cell lines. (B) The ellipse in the principal component analysis plot marks a 95% confidence interval. Partial separation of confidence intervals in PCA and objects clustering into 2 groups. (C) Western blot analysis for Skp2 expression of prostate cancer cell lines PC3 docetaxel-resistant (DR), PC3 age-control (AC), and mouse prostate adenocarcinoma E2 and cE2 cells with mesenchymal (DR, E2) and epithelial (AC, cE2) phenotypes. Representative results from three independent repetitions are shown. β-actin was used as a loading control.

Supplementary Figure 3. Proliferation in DU 145 mesenchymal and epithelial cells is equal but the size of tumorspheres and $CD44^{+}CD24^{-}$ CSC subpopulation is decreased in DU 145 epithelial cells. (A) Expression of CD24 and CD44 measured as the median fluorescence index (MFI) in DU 145 mesenchymal and epithelial cells using flow cytometry. Bars show the mean ± SD values for particular markers from three independent experiments. (B) qRT-PCR analysis of CD24 and CD44 expression. Data represent the mean ± SD of five independent experiments. The gene expression was normalized to *POLR2A* expression. (C) Quantification of $CD24^{+}$, $CD44^{+}$, and $CD44^{+}CD24^{-}$ subpopulations in mesenchymal and epithelial DU 145 cells by flow cytometry. Data are presented as the mean ± SD of the percentage of CD24 and CD44 positive cells, and $CD44^{+}CD24^{-}$ cancer stem-like cells. Results are from three independent repetitions. (D) Cell cycle analysis of epithelial and mesenchymal sublines of DU 145. Results are from three independent experiments obtained by using flow cytometry. (E) Tumorsphere formation rate in DU145 mesenchymal and epithelial cells. Bars show the mean ± SD. Tumorsphere formation rate was calculated as the number of tumorspheres divided by a number of seeded cells. Results are from three independent repetitions.

(F) Size of tumorspheres in DU 145 epithelial and mesenchymal cells. Results are from three independent repetitions.

Supplementary Figure 4. Decreased expression of Skp2 in accompanied with decreased tumorsphere formation rate, but not with changes in proliferation in mesenchymal DU 145. (A) Detection of $CD24^{+}$, $CD44^{+}$, and $CD44^{+}CD24^{-}$ cells in *SKP2* KD cell by flow cytometry. Data are presented as the mean ± SD of the CD24 and CD44 positive cells, and $CD44^{+}CD24^{-}$ cancer stem-like cells. Results are from three independent repetitions. (B) qRT-PCR analysis of CD24 and CD44 gene expression. Data represent the mean ± SD of five independent experiments. The gene expression was normalized to *POLR2A* expression. (C) Cell cycle analysis of control and Skp2 KD DU 145 cells. Data are from three independent experiments. (D) Tumorsphere formation rate in DU 145 mesenchymal control and *SKP2* KD cells. Bars show the mean ± SD. Tumorsphere formation rate was calculated as the number of tumorspheres divided by a number of seeded cells. Results are from three independent repetitions. (E) Size of tumorspheres in DU 145 mesenchymal control and *SKP2* KD cells. Results are from three independent repetitions.

Supplementary Figure 5. CD24 has decreased in DU 145 cells after Skp2 overexpression.
(A) Detection of Skp2 and p27Kip1 protein expression by western blot in control and Skp2-overexpressing DU 145. Representative images are from three independent experiments. α-tubulin was used as a loading control. (B) Percentage of $CD24^{+}$, $CD44^{+}$ cells and $CD44^{+}CD24^{-}$ subpopulation in control (empty vector) and Skp2 overexpressing DU 145 analyzed by flow cytometry. Data are presented as the mean ± SD of the percentage of $CD24^{+}$, $CD44^{+}$, and $CD44^{+}CD24^{-}$ cells. Results are from three independent experiments.

Supplementary Figure 6

Whole blot scans and uncropped membrane scans, related to western blots presented in this study.

Supplementary Table 1

List of primary and secondary antibodies used for western blot

Primary Antibody	Supplier	Source	Cat. No.	Dilution
α-tubulin	Sigma-Aldrich	Mouse	T9026	1:4000
β-actin	Sigma-Aldrich	Mouse	A5441	1:8000
E-Cadherin	BD Pharmingen	Mouse	610182	1:5000
N-Cadherin	BD Pharmingen	Mouse	610920	1:2500
$p27^{Kip1}$	Santa Cruz	Rabbit	SC-528	1:500
$p27^{Kip1}$	Cell Signaling	Rabbit	2552	1:1000
Skp2	Cell Signaling	Rabbit	4313	1:1000
Trop-2	R&D	Mouse	AF650	1:250
Trop-2	R&D	Goat	AF1122	1:250
vimentin	Sigma-Aldrich	Mouse	V6389	1:500
vimentin	Sigma-Aldrich	Rabbit	SAB300676	1:500
Zeb1	Bethyl	Rabbit	A301-922A	1:500
Zeb2	Bethyl	Rabbit	A302-474A	1:500

Secondary Antibody	Supplier		Cat. No.	Dilution
[HRP]-linked anti-rabbit IgG1,	Amersham	Sheep	NA934V	1:3000
HRP-linked anti-mouse IgG1	Amersham	Sheep	NA931	1:4000

Supplementary Table 2

List of primary antibodies, Isotype controls and probes used for for flow cytometry

**Antibody**	**Fluorochrome**	**Source**	**Supplier**	**Cat. No**	**Dilution**
Surface markers					
CD24	Briliant Violet 421	Mouse	BioLegend	311122	1:20
CD24	PE/Cy7	Mouse	BioLegend	311122	1:20
CD44	APC/Cy7	Rat	BioLegend	103 028	1:100
Isotype controls					
IgG2a	Briliant Violet 650	Mouse	BioLegend	711-126-152	1:20
IgG2a	PE/Cy7	Mouse	BioLegend		1:20
IgG2b	APC/Cy7	Rat	BioLegend	400624	1:100
Viability					
LIVE/DEAD® Fixable Dead Cell Stain Kit	Far Red	-	Thermo Fisher Scientific	L10120	1:1000

Supplementary Table 3
List of antibodies used for immunohistochemistry

Primary Antibody	Supplier	Source	Clone	Cat. No.	Dilution	Antigen retrieval Method
Skp2	Invitrogen	Mouse	2C8D9	18-7334	1:75	Ventana
E-cadherin	Dako	Mouse	NCH-38	IR05961-2	1:50	MW, citrate, pH 6.0
vimentin	Dako	Mouse	V9	IR63061-2	1:10	MW, citrate, pH 6.0

* Final detection was performed either with ultraView Universal DAB Detection Kit (Ventana, Roche) or EnVision® + Dual Link System-HRP (DAB+) (Dako/Agilent); MW, microwave

Supplementary Table 4
Clinical data related to patient samples

**prostate cancer patients**	**age at diagnosis**	**PSA total [ng/mL]**	**Gleason score**	**TNM**		**stage**	**risk**
patient 1	56	5,86	<7	pT2b	pN0	local	low
patient 2	60	14,70	<7	pT4	pN0	advanced	high
patient 3	66	20,40	<7	pT4	pN0	advanced	high
patient 4	55	3,66	<7	pT2b	pN0	local	low
patient 5	65	4,22	<7	pT2a	pN0	local	low
patient 6	51	6,01	<7	pT2b	pN0	local	low
patient 7	66	14,10	<7	pT3b	pN0	advanced	high
patient 8	65	4,40	<7	pT2b	pN0	local	low
patient 9	65	9,80	<7	pT2b	pN0	local	low
patient 10	69	n/a	<7	pT4	pN0	advanced	high
patient 11	70	17,40	<7	pT2b	pN0	local	low
patient 12	63	7,06	<7	pT2c	pN0	local	low
patient 13	55	8,35	<7	pT3b	pN0	advanced	high
patient 14	57	33,60	<7	pT3b	pN0	advanced	high
patient 15	62	3,59	<7	pT2c	pN0	local	low
patient 16	64	17,30	<7	T2c	pN0	local	low
patient 17	62	7,77	<7	T2c	pN0	local	low
patient 18	60	8,21	<7	pT2c	pN0	local	low
patient 19	56	7,60	<7	pT2c	pN0	local	low
patient 20	72	10,20	<7	pT3a	pN1	metastatic	high
patient 21	58	6,40	<7	pT2c	pN1	metastatic	high
patient 22	58	3,20	<7	pT2b	pNx	local	low
patient 23	65	21,50	7	pT3b	pN0	advanced	high
patient 24	70	n/a	7	pT4	pN0	advanced	high
patient 25	62	35,00	7	pT3b	pN0	advanced	high
patient 26	61	18,00	7	pT3b	pN0	advanced	high
patient 27	68	28,00	7	pT2b	pN0	local	high
patient 28	60	6,60	7	pT2b	pN0	local	low
patient 29	58	6,15	7	pT3b	pN0	advanced	high
patient 30	68	7,40	7	pT2a	pN0	local	low
patient 31	59	4,80	7	pT2b	pN0	local	low
patient 32	61	16,81	7	pT2b	pN0	local	low
patient 33	51	13,68	7	pT2b	pN0	local	low
patient 34	60	28,50	7	pT2b	pN0	local	high
patient 35	53	1,89	7	pT2a	pN0	local	low
patient 36	72	1,46	7	pT2a	pN0	local	low

patient 37	66	9,02	7	pT3b	pN0	advanced	high
patient 38	57	12,50	7	pT3b	pN0	advanced	high
patient 39	60	15,90	7	pT3a	pN0	advanced	high
patient 40	66	19,50	7	pT2c	pN0	local	low
patient 41	71	18,80	7	pT3b	pN0	advanced	high
patient 42	61	4,28	7	pT2a	pN0	local	low
patient 43	61	10,80	7	pT2c	pN0	local	low
patient 44	64	11,16	7	pT3b	pN0	advanced	high
patient 45	69	6,50	7	pT3b	pN0	advanced	high
patient 46	66	14,70	7	pT3b	pN0	advanced	high
patient 47	62	6,04	7	pT3b	pN0	advanced	high
patient 48	49	9,13	7	T2c	pN0	local	low
patient 49	66	7,17	7	pT2c	pN0	local	low
patient 50	51	9,64	7	pT2a	pN0	local	low
patient 51	66	3,11	7	pT2a	pN0	local	low
patient 52	50	8,30	7	pT2c	pN0	local	low
patient 53	65	6,70	7	pT2c	pN0	local	low
patient 54	52	3,90	7	pT2b	pN0	local	low
patient 55	64	5,13	7	pT2c	pN0	local	low
patient 56	73	11,99	7	pT2c	pN0	local	low
patient 57	64	n/a	7	pT2c	pN1	metastatic	high
patient 58	63	n/a	7	pT3b	pN1	metastatic	high
patient 59	56	n/a	7	pT3b	pN1	metastatic	high
patient 60	56	35,00	7	pT3b	pN1	metastatic	high
patient 61	56	12,70	7	pT3b	pN1	metastatic	high
patient 62	57	2,78	7	pT3a	pN1	metastatic	high
patient 63	62	15,44	7	pT4	pN1	metastatic	high
patient 64	61	25,46	7	pT2c	pN1	metastatic	high
patient 65	68	33,16	7	pT3b	pN1	metastatic	high
patient 66	59	40,00	7	pT3b	pN1	metastatic	high
patient 67	63	41,00	7	pT3b	pN1	metastatic	high
patient 68	63	10,30	7	pT3b	pNx	advanced	high
patient 69	62	8,53	7	pT3b	pNx	advanced	high
patient 70	68	6,29	7	pT3b	pNx	advanced	high
patient 71	63	9,80	7	pT2c	pNx	local	low
patient 72	69	4,00	>7	pT3b	pN0	advanced	high
patient 73	55	31,20	>7	pT3b	pN0	advanced	high
patient 74	57	12,12	>7	pT3b	pN0	advanced	high
patient 75	58	13,74	>7	pT4	pN0	advanced	high
patient 76	73	3,06	>7	pT3b	pN0	advanced	high
patient 77	55	3,60	>7	pT3b	pN0	advanced	high
patient 78	61	7,48	>7	pT3b	pN0	advanced	high
patient 79	67	6,05	>7	pT3b	pN0	advanced	high

patient 80	59	6,03	>7	pT3b	pN0	advanced	high
patient 81	74	5,20	>7	pT3b	pN0	advanced	high
patient 82	71	15,10	>7	pT3b	pN0	advanced	high
patient 83	64	n/a	>7	pT4	pN1	metastatic	high
patient 84	62	13,60	>7	pT3b	pN1	metastatic	high
patient 85	69	3,10	>7	pT3b	pN1	metastatic	high
patient 86	68	7,90	>7	pT3b	pN1	metastatic	high
patient 87	59	12,00	>7	pT3b	pN1	metastatic	high
patient 88	62	12,49	>7	pT2b	pN1	metastatic	high
patient 89	57	6,70	>7	pT2b	pN1	metastatic	high
patient 90	61	n/a	>7	pT4	pN1	metastatic	high
patient 91	63	9,37	>7	pT3b	pN1	metastatic	high
patient 92	60	9,51	>7	pT3b	pN1	metastatic	high
patient 93	67	n/a	>7	pT3b	pN1	metastatic	high
patient 94	56	28,00	>7	pT3b	pN1	metastatic	high
patient 95	64	12,20	>7	pT3b	pN1	metastatic	high
patient 96	76	30,77	>7	pT3b	pN1	metastatic	high
patient 97	70	11,00	>7	pT3b	pN1	metastatic	high
patient 98	69	10,30	>7	pT3b	pN1	metastatic	high
patient 99	66	8,29	>7	pT2b	pNx	local	low
patient 100	61	4,51	>7	pT3b	pNx	advanced	high
patient 101	66	11,66	>7	pT4		advanced	high

Supplementary Table 5

Clinical characteristics of the patients - summarization

Number of patients	101			
Age	49-60	61-70	71-76	
	36	57	8	
Serum PSA (ng/ml)	<4	4–10	>10	missing
	11	40	45	5
Gleason scores	<7	7	>7	
	22	49	30	
Cancer stages	pT2a-c	pT3a-b	pT4	
	42	50	9	
Lymph node status	pN1	pN0	pNx*	
	29	65	7	
*without lymphadenectomy or no information				

Supplementary Table 6

Human gene expression assays

Gene	Accesion #	F primer	R primer	Probe
CD24	NM_013230.2	TGGATTTGACATTGC ATTTGA	TGGGGGTAGATTCTC ATTCATC	#37
CD44	NM_000610.3	TCACATTAAGTTTGC ATGACCTG	AATAGGGCCAGCCTC TATGAA	#57
POLR 2A	NM_000937.3	CACGTCGACAGGAA CATCAG	GCAAATTCACCAAGAG AGACG	#1

Supplementary Figure 1

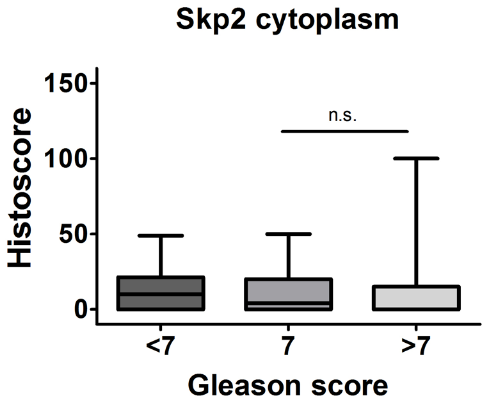

Supplementary Figure 2

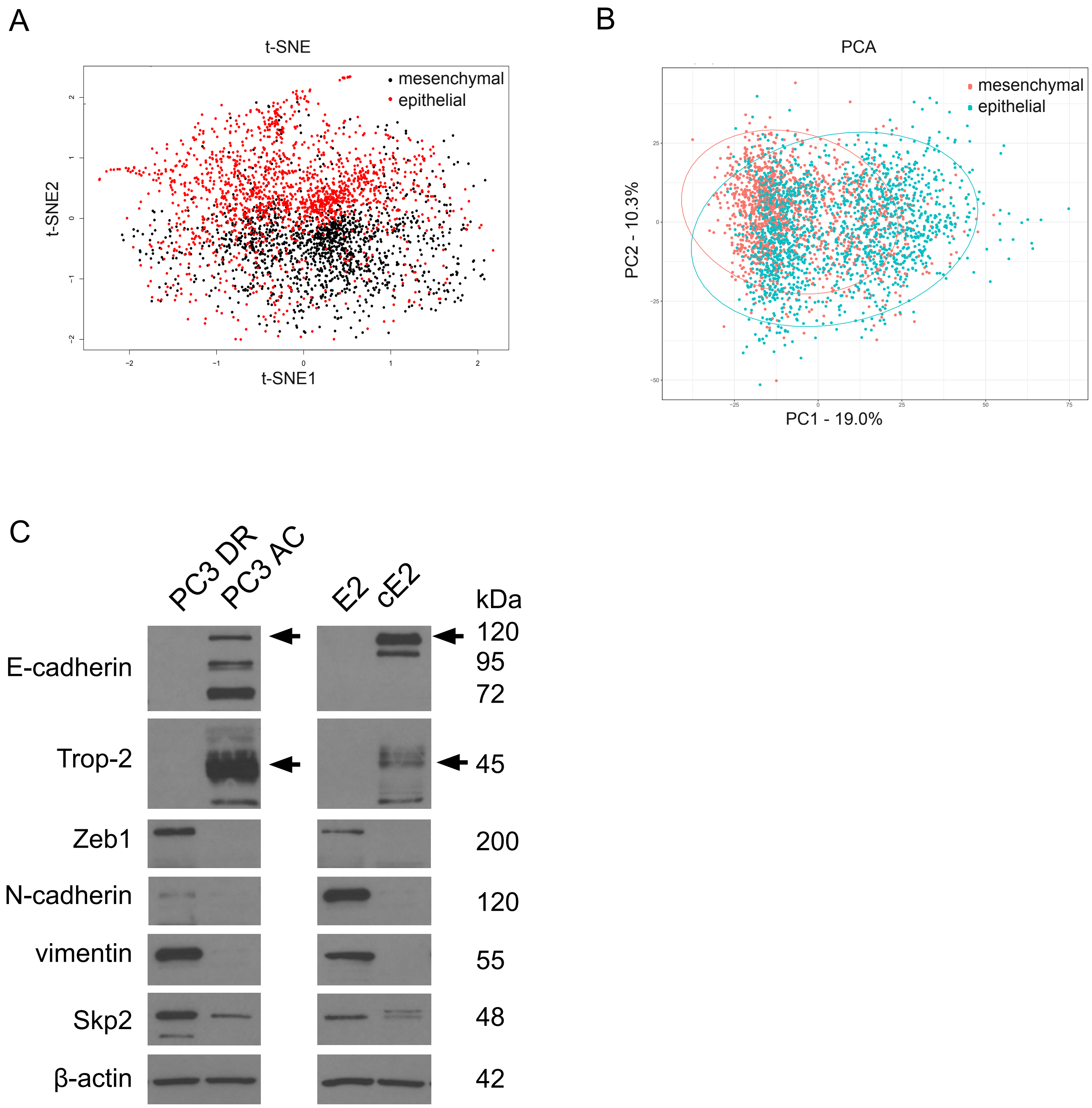

Supplementary Figure 3

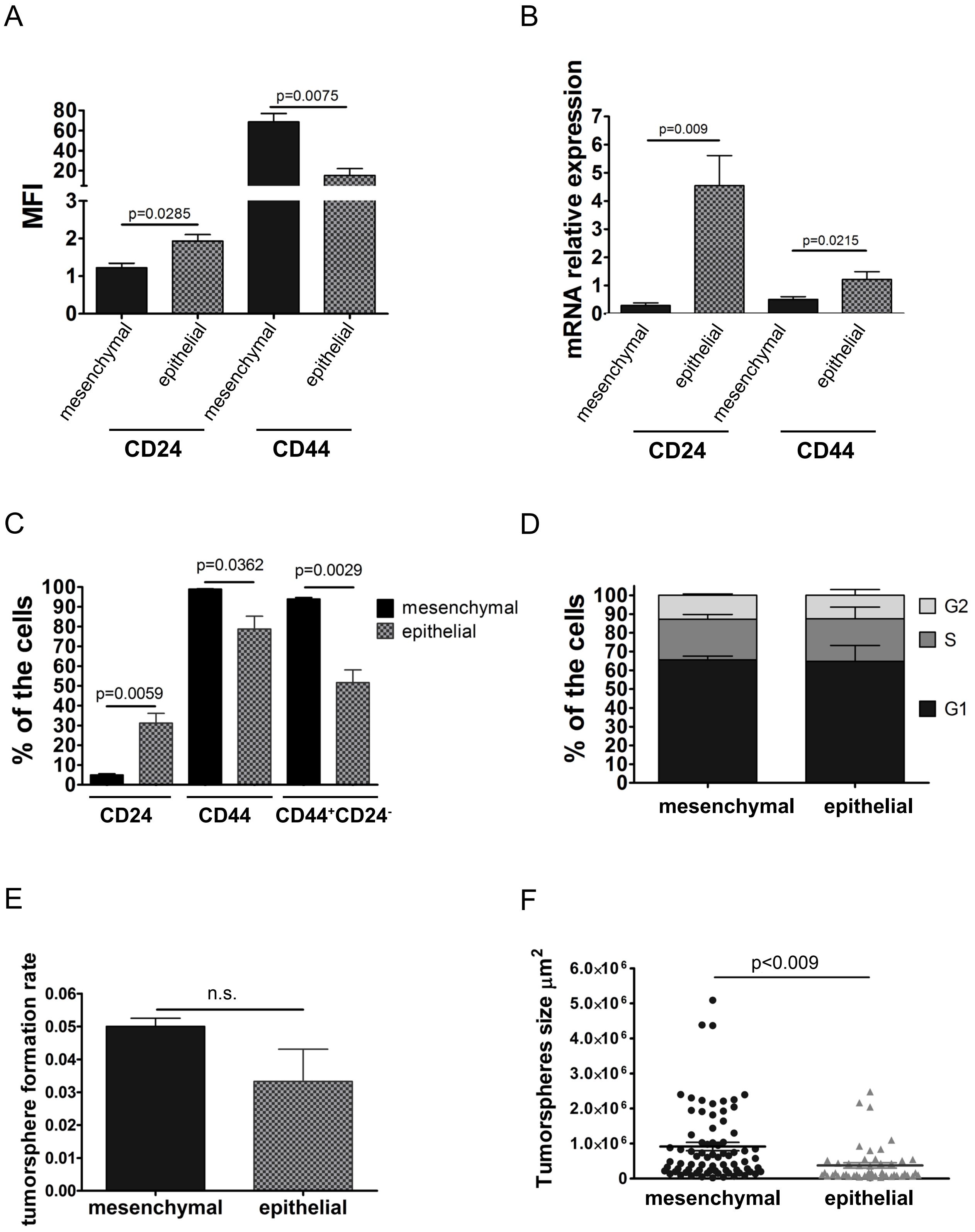

Supplementary Figure 4

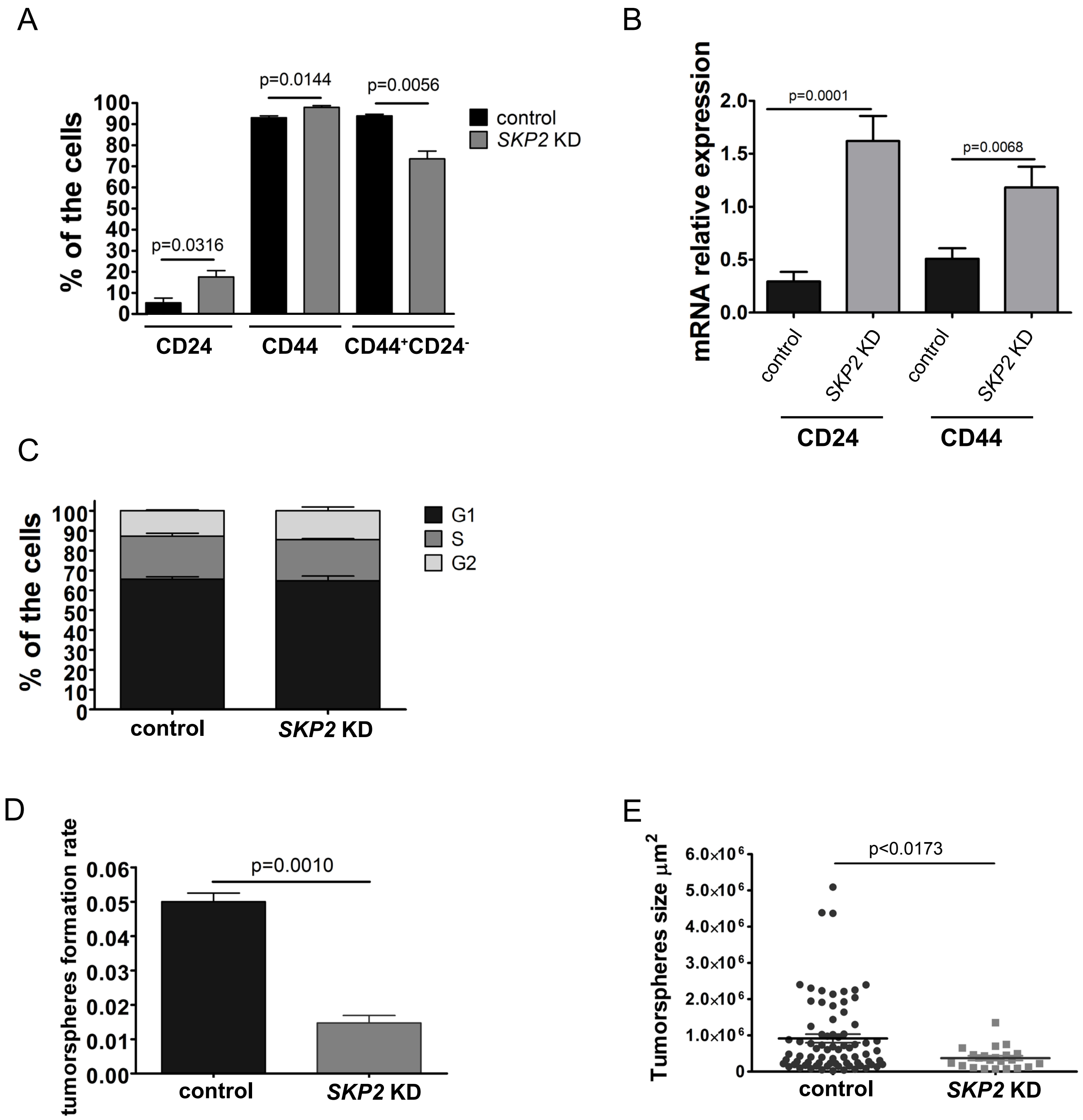

Supplementary Figure 5

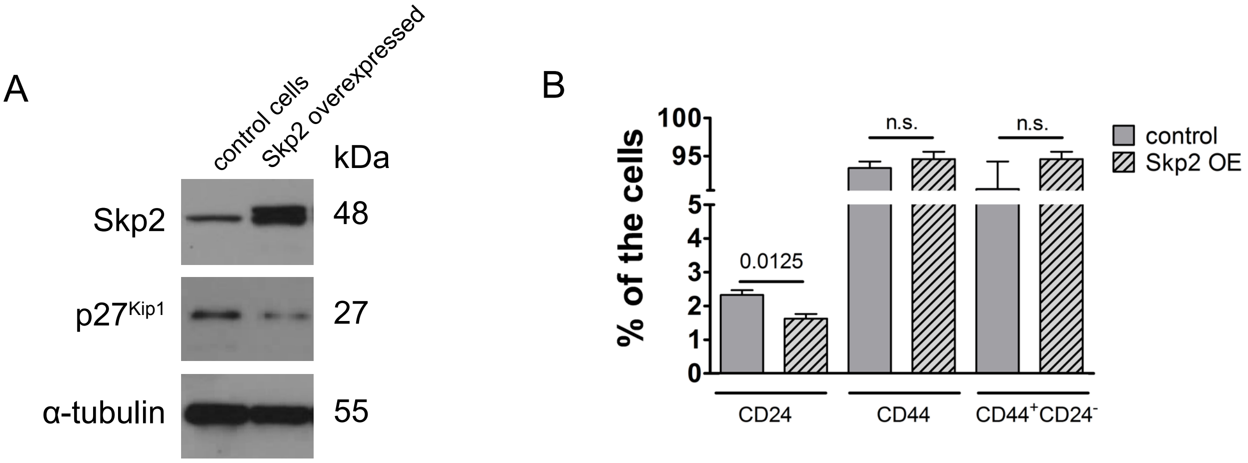

Supplementary Figure 6

Membranes related to Figure 2B

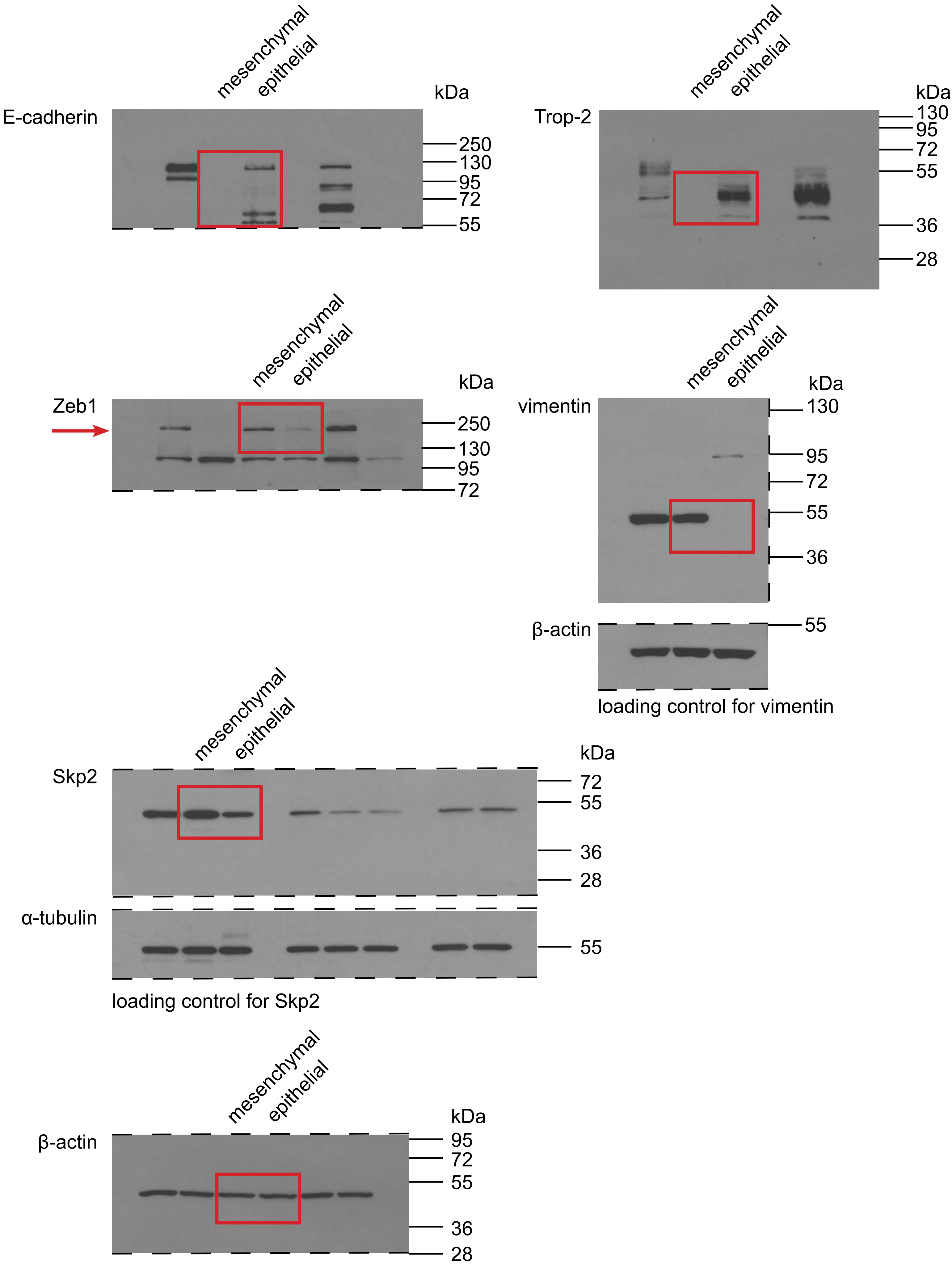

Supplementary Figure 6 cont.
Membranes related to Figure 4A

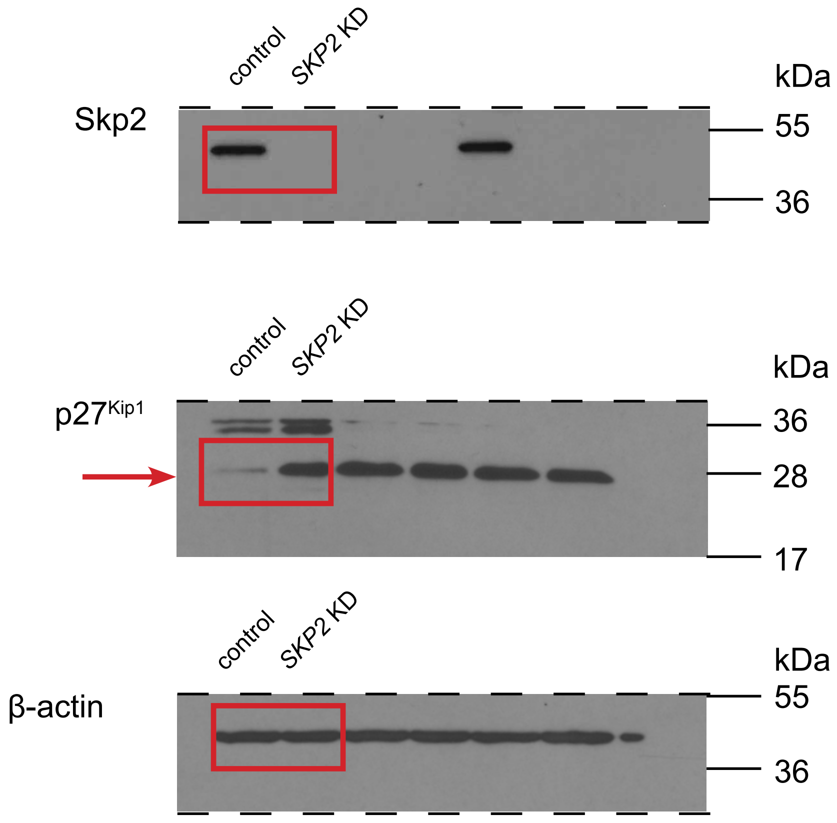

Supplementary Figure 6 cont.
Membranes related to Supplementary Figure 2C

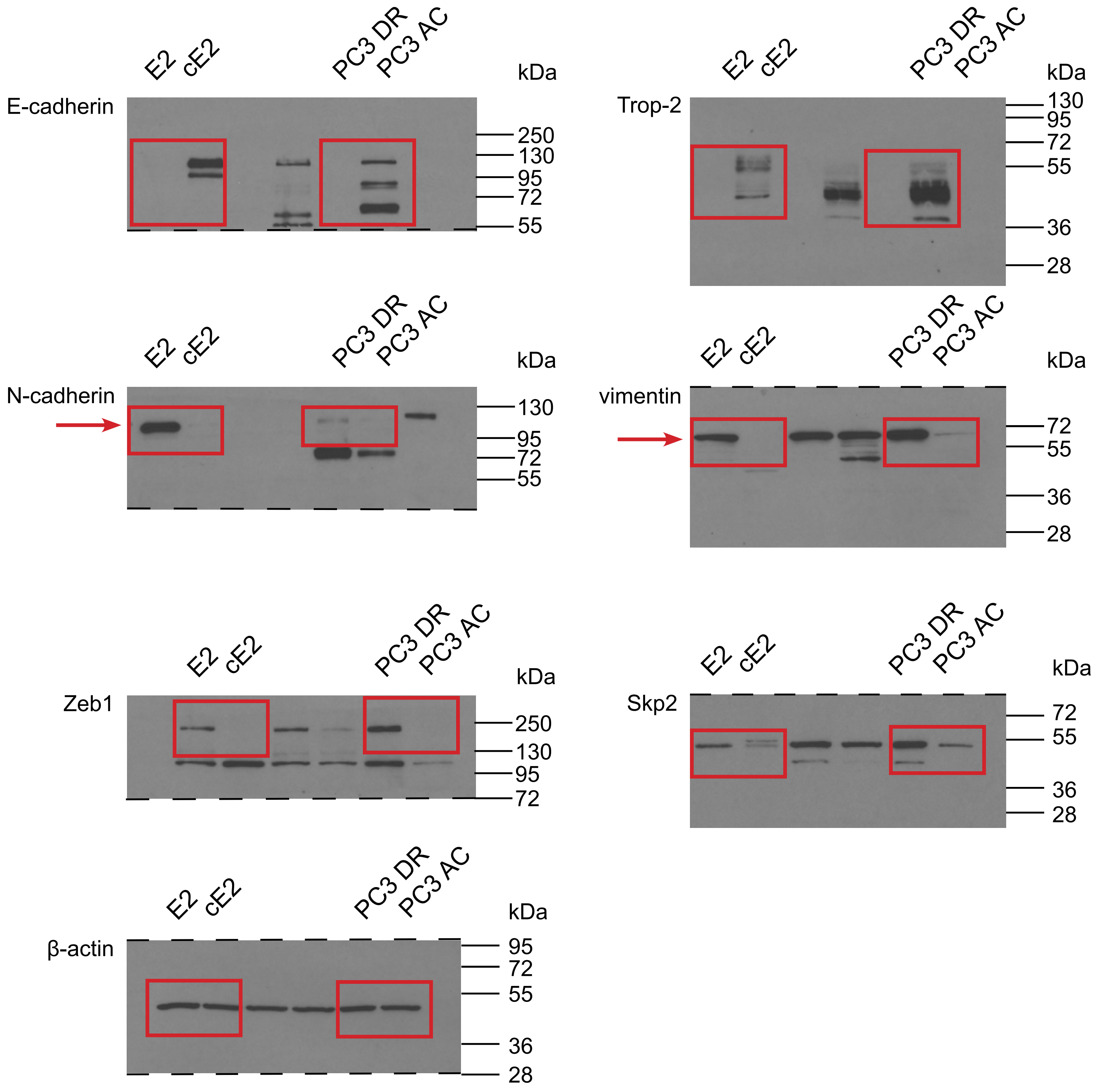

Supplementary Figure 6 cont.

Membranes related to Supplementary Figure 5A

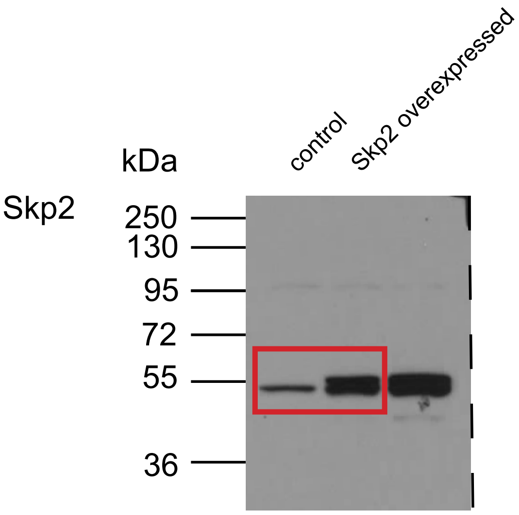

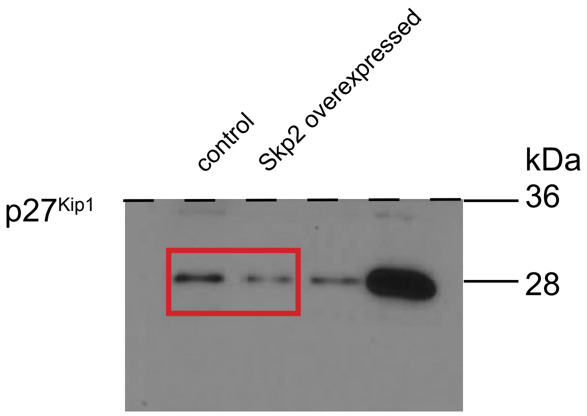

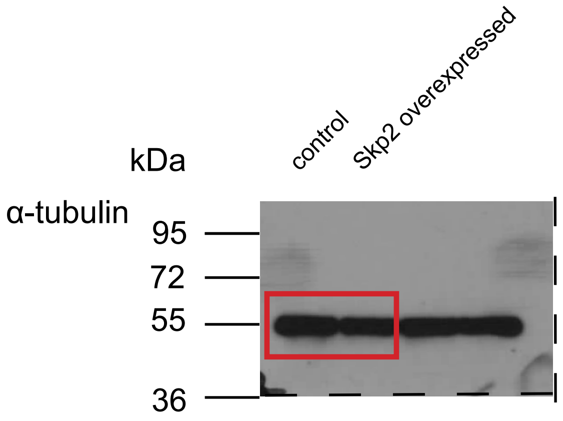

Supplement: Supplementary file 1 — Supplementary Materials [file 41598_2019_42131_MOESM1_ESM.pdf]
